# Protist size-dependent shifts of bacterial communities can reduce litter decomposition

**DOI:** 10.1093/ismeco/ycaf231

**Published:** 2025-12-06

**Authors:** Yuxin Wang, Justine D M Lejoly, Alejandro Berlinches de Gea, Sven van den Elsen, G F (Ciska) Veen, Stefan Geisen

**Affiliations:** Laboratory of Nematology, Wageningen University & Research, Droevendaalsesteeg 1, Wageningen, 6708PB, The Netherlands; Department of Terrestrial Ecology, Netherlands Institute of Ecology (NIOO-KNAW), Droevendaalsesteeg 10, Wageningen, 6708 PB, The Netherlands; Laboratory of Nematology, Wageningen University & Research, Droevendaalsesteeg 1, Wageningen, 6708PB, The Netherlands; Laboratory of Nematology, Wageningen University & Research, Droevendaalsesteeg 1, Wageningen, 6708PB, The Netherlands; Department of Terrestrial Ecology, Netherlands Institute of Ecology (NIOO-KNAW), Droevendaalsesteeg 10, Wageningen, 6708 PB, The Netherlands; Laboratory of Nematology, Wageningen University & Research, Droevendaalsesteeg 1, Wageningen, 6708PB, The Netherlands

**Keywords:** litter decomposition, protist size, microbial respiration, bacterial community

## Abstract

Microbial-mediated litter decomposition drives carbon and nutrient cycling. This process can be top-down regulated by microbiome predators, particularly the diverse protists. Size has been suggested to determine predation impacts, but how protists of different size categories affect microbial-mediated litter decomposition remains unknown. Using a litter decomposition experiment with three protist size categories, we investigated protist size-dependent effects on microbial-driven litter decomposition. We found that protists of the large-size category created more structurally similar bacterial communities compared to the no-protist control. These protists of the large size category also reduced litter mass loss by 40%, while increasing microbial respiration by 17% compared to the no-protist control after five weeks of decomposition. In contrast, protists of the small-size category and protists of the medium-size category had no measurable impact on bacterial communities, litter mass loss, or microbial respiration. Random forest analysis identified *Streptomyces* as a major contributor to litter mass loss (explained 8% of litter mass), while the potential protist symbionts *Taonella* and *Reyranella* explained 8% and 6% of microbial respiration, respectively. These likely predation-resistant bacterial taxa were primarily enriched by protists of the large-size category. Our results indicate that protists, especially large ones, can alter litter decomposition by shaping microbiome composition. Future studies on litter decomposition and carbon cycling should incorporate protists and their traits, particularly size, to enhance our understanding of global carbon and nutrient cycling.

## Introduction

Litter decomposition contributes to nutrient cycling [1] and soil organic matter (SOM) formation [2]. This process can function as either a carbon sink or source, depending on the balance between SOM accumulation and carbon dioxide release [3, 4]. In agroecosystems, the litter decomposition process is becoming increasingly important, as cover crop residues and compost are commonly applied to control pathogens, enhance water and nutrient availability [5–7], and increase carbon stocks in the soil [8, 9]. In addition to abiotic factors like climate and litter quality, soil decomposer communities determine litter decomposition rates [10–12].

Bacteria and fungi are the primary decomposers in soil ecosystems [13], their precise contributions to litter decomposition vary depending on multiple factors such as pH, moisture, and substrate composition [14]. While fungi are commonly regarded as the dominant decomposers [15], bacteria can be the primary decomposers in certain substrates, particularly in nutrient-rich or easily degradable materials, and also play a crucial role in the early stages of decomposition [16]. In addition to their greater efficiency in assimilating simple organic compounds compared to fungi [17], certain bacterial groups, such as Proteobacteria (e.g. *Rhizobiales*), Actinobacteria (e.g. *Streptomyces*), and Firmicutes (e.g. *Bacillus*), are known for producing diverse extracellular enzymes that contribute to the breakdown of recalcitrant carbon sources, making them crucial players in litter decomposition [16, 18–21]. Beyond their capacity for direct litter degradation, bacteria also affect litter decomposition through mutualistic and competitive interactions with fungi [17, 22]. Some bacteria can facilitate fungal decomposition by providing access to organic nitrogen [23], while others, such as *Streptomyces,* can inhibit fungal activity by producing antibiotics [24].

Bacteria are not acting in isolation, but are top-down controlled by protists as dominant bacterivores [25]. Arguably, the impact of protists on bacterial communities is less evident at the level of alpha diversity, as most protists and their combinations are not shown to affect bacterial alpha diversity, with only a few cases suggesting protist-induced increases [26–28]. In turn, by feeding on a wide range of bacterial species with some feeding selectivity based on prey traits, such as digestion resistance, cell wall structure, and motility [29], protists generally determine bacterial community composition and function [30, 31]. In response, bacterial communities shift toward predation-resistant taxa [32], such as digestion-resistant Gram-positive bacteria [33–35]. This shift is linked to protist-induced shifts in bacterial functioning, such as decreasing hydrocarbon degradation [36]. These shifts in bacterial functioning may influence litter decomposition, with studies reporting inconsistent effects. For instance, litter decomposition and CO_2_ release were reported to increase in a study using *Physarum polycephalum* as a model [37], while other studies found that protists did not affect litter decomposition (i.e. with the addition of *Didymium* sp. and *Cercomonas ambigua*), and even led to a reduction in litter decomposition (with *Tetramitus thorntoni*) [38]. Generally, the underlying mechanisms of protist-induced changes in litter decomposition remain to be determined.

Ecophysiological traits such as size are key factors influencing how predators, such as protists, affect shifts in bacterial communities, with larger protists generally exerting stronger effects than smaller protists [39]. Larger size is typically associated with higher metabolic rates and feeding demands [40], as well as greater mobility, which increases the likelihood of encountering prey [41, 42]. Together, these traits likely intensify predation pressure on bacterial communities, potentially amplifying their effects on bacterial community composition. These changes, such as increasing Gram-positive relative to Gram-negative bacteria, may lead to alterations in bacterial functional processes, such as causing a greater reduction in hydrocarbon degradation, which could further reduce overall litter decomposition. However, it remains unknown whether protist-predation-induced shifts in bacterial communities underlie changes in litter decomposition and whether effects increase with protist size.

To investigate whether the effects of protists on bacterial communities scale with protist size and whether these bacterial shifts correspond to changes in litter decomposition, we selected 12 bacterivorous protists and grouped them into three size categories: small, medium, and large. To minimize species-specific effects within each size category, we randomly selected three out of four species to assemble protist communities. This design enabled a species-independent analysis of the impact of protist size on bacterial communities, litter decomposition, and microbial respiration, over a 35-day litter decomposition experiment. We hypothesized that protist effects increase with size category, leading to: (i) shifts in bacterial community composition, such as enhancing predation-resistant Gram-positive bacteria and reducing carbon-degrading functional groups, with bacterial diversity remaining unaffected; (ii) decreases in litter decomposition and microbial respiration. Furthermore, we expected that (iii) changes in bacterial community composition (e.g. dominance of Gram-positive bacteria and suppression of carbon-degrading functional groups) could explain variations in litter decomposition and microbial respiration.

## Materials and methods

### Litterbag preparation

We used three cover crop mixtures from Vredepeel farmland in the southeast of the Netherlands (51°32′27"N 5°50′59"E) as litter substrates. These cover crop species were fescue (C/N = 13.39), marigold (C/N = 47.05), and oats (C/N = 36.85). We cut shoots, oven-dried them, and chopped dried material into 1–3 cm fragments. After thorough homogenization and mixing, we added 2 g of these cover crop mixtures into 250 μm mesh bags (7 cm × 7 cm). The initial carbon quality of this composite mixture was determined as the average of the C/N ratios of the three cover crops (C/N = 32.43). All litterbags were stored at room temperature (~20°C) and thermal-sterilized in an oven at 70°C for 72 h [43] before being buried in the soil.

### Protist size measurement

We measured the maximum length and width of at least 10 active protist individuals per species (see Supplementary Information 1.1 for details on protist inoculum preparation) under 400x magnification using a Zeiss Axioskope 2 Plus (Zeiss, Germany). However, the choice of the parameter to represent the size of protists remains a subject of debate. Researchers have explored various methods to quantify protist size: (i) measuring the volume of the cyst or the nuclear diameter [44]; (ii) measuring maximum length; (iii) using the maximum length and width to calculate the volume from the closest geometric shape [39, 45]; (iv) using the maximum length and width to calculate the area size. Each method has its limitations: method 1 is limited by the fact that not all protists form cysts (such as *Vannella* spp. in our cultures); method 2 is constrained by considering only one dimension; method 3 introduces larger biases by using two observations to calculate the 3D volume of an irregularly shaped amoeba, as it requires assumptions about the object's shape and mass distribution, potentially leading to inaccuracies. After comparing the limitations of these measurement methods, we chose the area size of the observed protists under the microscope as the parameter to determine their size. This approach minimizes biases associated with estimating 3D volume and addresses variations caused by cultures that cannot form cysts. For more details, see Table S1.

### Experimental design

We conducted a 35-day microcosm decomposition experiment to investigate the impact of protist size on bacterial communities, litter decomposition, and microbial respiration. Twelve bacterivorous protists were selected (see Supplementary Information 1.1 for details on protist inoculum preparation) and categorized into three size categories: small, medium, and large (Table S2). These protist species were chosen because (i) they represent widespread bacterivorous taxa commonly occurring in soils and litter residues, and (ii) they span different size categories and are evenly distributed across larger protist lineages, minimizing the influence of phylogenetically conserved traits (e.g. enzymes) (Table S1). Although protists may show feeding preferences (e.g. tending to consume Gram-negative bacteria more efficiently than Gram-positive bacteria), all are generalist bacterivores capable of consuming a wide range of bacteria [39, 46]. To assemble protist communities within each size category, we randomly selected three out of four species, ensuring a species-independent analysis while enhancing the ecological realism of the experiment by capturing functional diversity within size categories (see Supplementary Table S2 for details on inoculation). This experiment included a no-protist control (referred to as P-) and three protist size treatments: protists of the small-size category (referred to as Ps), protists of the medium-size category (referred to as Pm), and protists of the large-size category (referred to as Pl). Each treatment, including the control, was replicated 10 times. A total of 200 g of gamma-sterilized grassland soil from the Veluwe area (52°03′38.1"N 5°45′10.6"E; De Mossel, Ede, the Netherlands) was placed into 40 plastic pots (10 cm × 10 cm × 5 cm). The prepared polyamide litterbags containing cover crop mixtures were buried under the soil surface to simulate natural conditions. After initial watering with 20 ml of demineralized water, all 40 microcosms were inoculated with 2.4 ml of a homogenized and standardized microbiome suspension, consisting of bacterial and fungal inocula washed and resuspended in Neff’s Modified Amoebae Saline (NMAS) to remove excess nutrients (see Supplementary Information 1.2 & 1.3). 8 days after inoculation, microbiome establishment was assessed via CO_2_ respiration measurements, which showed no significant differences among treatments (data not shown). Two days after assessing the microbiome establishment, we inoculated the Ps, Pm, and Pl treatments with a suspension of protists of the small-size category, medium-size category, and large-size category, respectively (washed and resuspended in NMAS; see Supplementary Information 1.1 for details), while the no-protist control (P−) received an equal volume of NMAS to balance water content across treatments. This resulted in 20 000–30 000 protist cells in each microcosm containing protists (Table S2), where this variation came from the adjusted concentration of different protist species, as some species were initially present at lower concentrations than others. During incubation, containers were placed in a dark climate chamber with air moisture content at 70% and temperature between 15°C at night (8 h) and 20°C during the daytime (16 h). Moisture content was monitored and maintained at 20% wt by adding demineralized water once a week.

### CO_2_ flux and litter mass loss measurement

To assess carbon loss through microbial respiration, we monitored CO_2_ emissions during incubation. Initially, we determined CO_2_ once two days before protists' inoculation (day 0) to ensure treatment homogenization. Subsequently, we measured CO_2_ twice per week after protist inoculation on days 3, 6, 10, 13, 17, 20, 24, 27, 30, and 33. We sampled the headspace after a 2-h incubation using Exetainer vials (Labco, Lampeter, United Kingdom). The concentration of CO_2_ was measured on a CH_4_/CO_2_ analyzer GC-Trace1600-TCD (Thermo Fisher Scientific). We calculated cumulative CO_2_ emissions by assuming that the time between the samples was similar to the average of both samples: Cumulative C released as CO_2_ (${\int}_i^j{\varphi}_T$) =${\varphi}_{T_i}+\frac{\varphi_{T_i}+{\varphi}_{T_j}}{2}\ast{T}_{j-i}/2$, where $\varphi$ is the flux at time point T and ${T}_{j-i}$ is the time between the different time points (i and j). While we cannot separate bacterial from protist respiration in our study, previous studies revealed that the contribution of protists to overall respiration is negligible compared to that of bacteria and fungi, representing ca. 6% of total ecosystem respiration [47], and even less based on stable isotope labeling data [48]. Observed differences in the cumulative respiration among treatments, therefore, mainly derive from protist-driven shifts in bacterial communities, which can double microbial respiration [49, 50].

After five weeks of incubation, we harvested all litterbags and divided each sample into two parts. Around 0.25 g of fresh subsamples were weighed before and after oven-drying at 70°C for 72 h to calculate the water content as follows: $Water\ content=\frac{Weight_{fresh}-{Weight}_{dry}}{Weight_{dry}}$ and the mass loss is calculated as follows: $Mass\ loss= Initial\ {Weight}_{dry}-\frac{Final\ {Weight}_{fresh}}{1+ Water\ content}$. The remaining fresh subsamples were stored in a −20°C freezer for DNA extraction.

### DNA extraction and amplicon sequencing

We extracted DNA from litter material following an adapted protocol developed by Harkes *et al.* [51]. Briefly, 0.25 g of mixed fresh litter underwent bead beating, humic acid precipitation with ammonium aluminum sulfate, and phenol-chloroform extraction. The DNA was stored at −20°C until further use.

We sent the DNA for sequencing bacterial communities at Genome Quebec (Quebec, Canada). There, we amplified the V4-V5 region of the 16S ribosomal RNA gene using the standard primers 515F-Y (5′-GTGYCAGCMGCCGCGGTAA-3′) and 926R (5′-CCGYCAATTYMTTTRAGTTT-3′) [52]. Amplicons were sequenced on an Illumina NextSeq 2000 platform (Illumina, San Diego, USA) using a P1 flow cell, generating 2 × 300 bp paired-end reads. The run lasted 34 h, with an average Q score of 32 and over 97% of bases above Q30. After several trials to optimize read retention, we processed the sequencing data using DADA2 [53]. Both forward and reverse primers were trimmed starting at 10 bp, and reads were truncated at 220 bp, using the filterAndTrim function, yielding 2 × 210 bp fragments. Reads with fewer than five expected errors (maxEE = 5) were retained, while other filtering parameters (e.g. maxN, truncQ) were left at default. Chimeric sequences were identified and removed using the consensus method implemented in DADA2 [54]. The remaining high-quality reads were then processed for error correction, denoising, and paired-end merging to infer true biological sequences, generating Amplicon Sequence Variants (ASVs). After DADA2 processing, the dataset retained a total of 877 206 high-quality reads across all samples (minimum = 11 016 reads/sample; median = 22044.5 reads/sample). We assigned taxonomy to each ASV using the scikit-learn method [55] with a Naive Bayes classifier trained specifically for our primer pairs via the Reference Sequence Annotation and Curation Pipeline (RESCRIPt) [56], based on the SILVA v138.1 database. The resulting ASV abundance table and taxonomic classifications were used for downstream analyses. Additionally, ASV data were rarefied to 10 000 reads per sample before downstream analyses to account for differences in sequencing depth while ensuring that all samples were preserved.

Subsequently, bacterial ASVs were functionally annotated using FAPROTAX (Functional Annotation of Prokaryotic Taxa) as implemented in the *microeco* package [57, 58], focusing on key bacterial processes involved in carbon and nitrogen cycling. We note that using 16S rRNA gene data to infer function through FAPROTAX cannot provide definitive measurements of microbial activity, but offers a general indication of the potential functional capacities linked to functions performed by cultured relatives [57]. While FAPROTAX cannot resolve all functions in full depth, we consider it to provide more reliable insights into potential bacterial functions in soils compared to genome-inferred tools (e.g. Tax4Fun, PICRUSt), which are highly biased towards human-obtained information. In this sense, FAPROTAX is conceptually comparable to routinely used fungal annotation tools such as FUNGuild or FungalTraits [59, 60]. Specifically, the carbon-cycling bacterial functional groups included methylotrophy, fermentation, hydrocarbon degradation, and chemoheterotrophy, while the nitrogen-cycling bacterial functional groups encompassed nitrogen fixation, nitrogen respiration, and ureolysis. Notably, nitrogen fixation and ureolysis contribute to nitrification, whereas nitrogen respiration is associated with denitrification. To ensure a biologically relevant functional analysis, we applied a filtering threshold of 0.1% average abundance, which led to the exclusion of cellulolysis. Additionally, we incorporated the “predatory or exoparasitic” functional group due to the similarity in function with protists and the resulting ecological role in shaping bacterial community composition. Beyond functional annotation, we also calculated the Gram-positive to Gram-negative bacterial ratio to gain insights into community composition and potential functional differences, particularly concerning predation resistance. For this classification, we categorized *Actinomycetota* and *Bacillota* as Gram-positive bacteria. The following phyla were classified as Gram-negative: *Abditibacteriota*, *Acidobacteriota*, *Armatimonadota*, *Bacteroidota*, *Bdellovibrionota*, *Chloroflexota*, *Cyanobacteriota*, *Thermodesulfobacteriota*, *Gemmatimonadota*, *Myxococcota*, *Planctomycetota*, *Pseudomonadota*, and *Verrucomicrobiota*.

### Statistical analysis

To examine the effects of protist size on bacterial communities, litter decomposition, and microbial respiration, we categorized protist communities into three discrete size categories (small, medium, and large) rather than treating size as a continuous variable. This approach allowed for categorical comparisons and accounted for size category-specific differences, as each size category comprised 10 distinct protist communities (Table S2), resulting in compositional differences that did not follow a fully continuous gradient, making linear analysis unsuitable.

For datasets on litter mass loss, the relative abundance of bacterial taxonomy, the relative abundance of FAPROTAX-annotated bacterial functional groups and the Gram-positive to Gram-negative bacteria ratio that met the assumptions of normality and homogeneity, a one-way analysis of variance (ANOVA) was performed, followed by Tukey’s Honest Significant Difference (Tukey’s HSD) test (R base *stats* package) for pairwise comparisons. When assumptions were violated, the nonparametric Kruskal-Wallis test was employed, followed by Dunn’s test implemented in the *fsa* package [61] for *post hoc* pairwise comparisons. Bonferroni corrections were made to control Type 1 error rates among multiple tests [62]. We analyzed cumulative respiration across protist size categories using two complementary approaches. First, we fitted asymptotic regression curves with nonlinear mixed-effects models in the *nlme* package of R [63], following the method described in Bock and Wickings [64]. This modeling approach provides three biologically meaningful parameters: ɸ1, the maximum CO_2_ accumulated during incubation; ɸ2, the estimated starting respiration value at Day 0; and ɸ3, a rate constant describing how quickly respiration slowed over time. Protist treatments were included as fixed effects, while mesocosm identity was treated as a random factor. Model selection was based on Akaike Information Criterion (AIC; [65]), and differences between nested models were tested with likelihood ratio tests. Pairwise contrasts among treatments were then evaluated using the *emmeans* package [66]. Second, to assess the temporal dynamics of treatment effects, we performed a repeated-measures ANOVA on cumulative respiration across incubation days, followed by Tukey-adjusted *post hoc* pairwise comparisons (*emmeans* package). This allowed us to determine the time points at which protist size categories significantly diverged from the no-protist control (P−).

Alpha diversity metrics (Chao1 and Shannon indices) were calculated to assess bacterial richness and evenness among treatments. Principal Coordinate Analysis (PCoA) and Bray–Curtis distance analysis were used to evaluate differences in bacterial community composition. Both analyses were followed by permutational multivariate analysis of variance (PERMANOVA) utilizing the *adonis* function (999 permutations) from the R package *vegan* [67]. Pairwise Bray–Curtis distance comparisons between treatments were further analyzed using the Wilcoxon Rank Sum Test to identify significant differences. To identify bacterial indicator taxa associated with protists of different size categories, we employed three complementary approaches: (i) identification of the top 10 phyla and 40 genera based on relative abundance to characterize the dominant members of the bacterial community; (ii) Linear Discriminant Analysis Effect Size (LEfSe) with an LDA score threshold of 3.5 (α = 0.01) using the *microeco* package [58], to detect taxa that discriminated among protist size-category treatments; and (iii) differential abundance analysis (DAA) with the ANCOM-BC2 method implemented in the *file2meco* package [68] to identify taxa that differed significantly between each protist treatment and the no-protist control (*P <* .05).

To identify key predictors (top 10) of litter mass loss and cumulative respiration, we performed random forest (RF) analysis using the *rfPermute* package [69] with 1000 permutations and 500 trees. Predictors included litter water content, litter C/N ratio, bacterial PCoA parameters, bacterial Chao1 and Shannon indices, and the relative abundance of key bacterial groups. Specifically, we considered the relative abundance of the top 10 bacterial phyla, top 40 genera, and additional taxa identified from the LEfSe analysis, the FAPROTAX-annotated bacterial functional groups, and the Gram-positive to Gram-negative bacterial ratio. Percentage increases in the mean squared error (MSE) of variables were used to estimate the importance of these predictors, with higher MSE% values indicating more important predictors. All statistical analyses were performed using R software (version 4.1.0, R Core Team, 2020), and all graphs were made with the *ggplot2* package [70].

## Results

### Size effect of protists on bacterial diversity and community composition

**Figure 1:**
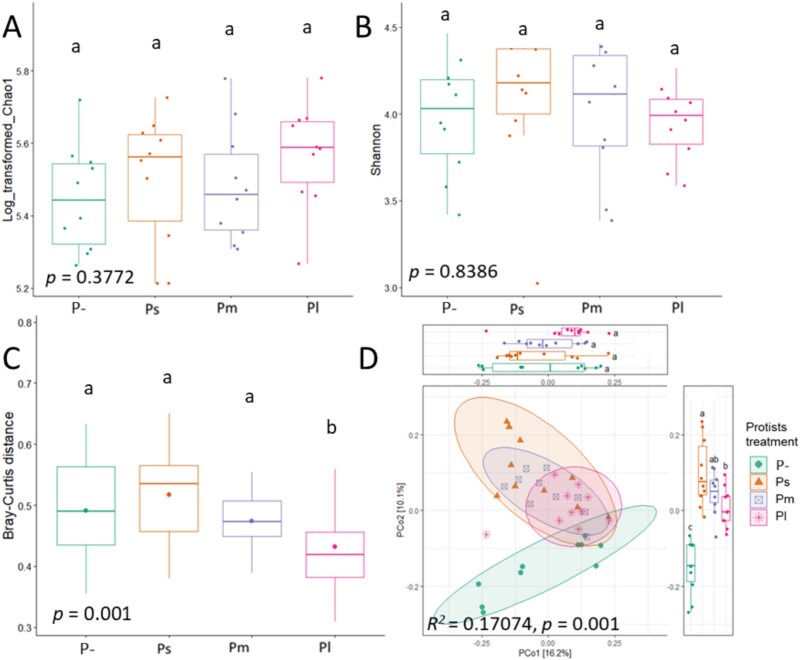
Size effects of protists on bacterial community composition. Effects of protist size on α-diversity Chao1 (A), Shannon index (B), Bray–Curtis distances of bacterial communities (C) and β-diversity of bacterial community analyzed via PCoA (D). P- = no-protist control, Ps = protists of the small-size category treatment, Pm = protists of the medium-size category treatment, Pl = protists of the large-size category treatment. In panels A, B and D, horizontal bars within boxes represent the median, with the tops and bottoms of boxes indicating the 75th and 25th quartiles, respectively. Whiskers depict the range of non-outlier data values, while outliers are plotted as individual points. Differences are evaluated by a one-way ANOVA test (*P <* .05), with different letters above bars indicating significant differences tested through Tukey’s HSD *post hoc* test (*P <* .05). In panel C, the significance of bacterial community dissimilarities among different treatments was assessed using PERMANOVA (*P <* .05), followed by pairwise Wilcoxon rank sum tests for *post hoc* comparisons, with p-values adjusted using the Bonferroni correction.

Protist size categories did not differentially affect bacterial diversity based on the Chao1 index (ANOVA; *F* = 1.06, *P* = .377; Fig. 1A) and the Shannon index (ANOVA; *F* = 0.281, *P =* .839; Fig. 1B). However, bacterial community dissimilarity increased with protist size category according to Bray–Curtis distance (PERMANOVA; R^2^ = 0.171, F = 2.47, *P =* .001; Fig. 1C and D). Pairwise comparisons showed differences in bacterial Bray–Curtis distance between no-protist control (P-) and protists of the large-size category (Pl) (*P =* .001), protists of the small-size category (Ps) and protists of the medium-size category (Pm) (*P =* .002), Ps and Pl (*P <* .001), and Pm and Pl (*P <* .001), while no significant differences were observed between P- and Ps or P- and Pm (Wilcoxon Rank Sum Test). Despite shifts in bacterial community composition, protist size categories did not differentially alter the ratio of Gram-positive to Gram-negative bacteria (Kruskal-Wallis, *P =* .404; Fig. S1) or the relative abundance of most FAPROTAX-annotated bacterial functional groups (Fig. S2), except for predatory bacterial functional group, which were more abundant in Ps compared to P- (Dunn’s test, *P =* .019; Fig. S2H), with no effects observed across other protist size categories.

### Size effect of protists on bacterial relative abundance and indicator taxa

**Figure 2:**
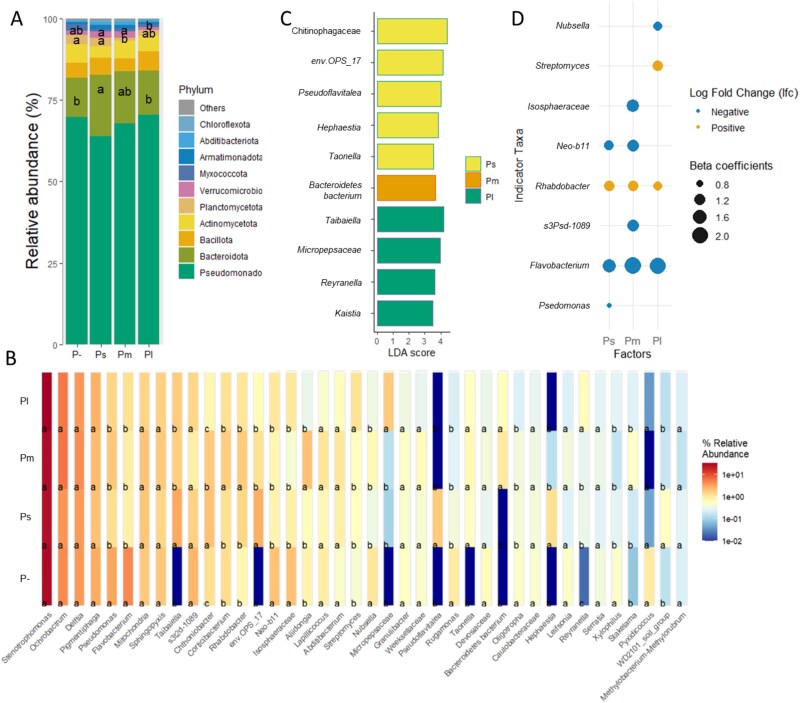
Size effects of protists on bacterial relative abundance and indicator taxa. Effects of protist size on bacteria community composition at the phylum level (A) and genus level (B). Representative bacteria among protists of different categories were selected based on linear discriminant analysis effect size (LEfSe) with an LDA threshold of 3.5; only the lowest taxonomy level of selected bacteria is shown (C). Differentially abundant bacterial taxa (at genus level) in each protist treatment compared to the no-protist control (P−), identified by ANCOM-BC2 (*P* < .05). Dot size corresponds to the beta coefficient from ANCOM-BC2. In panel C, the genus level of bacteria is shown in italics, the family level is in non-italic with regular font. P- = no-protist control, Ps = protists of the small-size category treatment, Pm = protists of the medium-size category treatment, Pl = protists of the large-size category treatment. Significant differences between different treatments in panels A and B are evaluated by a one-way ANOVA test (*P* < .05), with different letters above bars (only significant results are labeled) indicating significant distinctions tested through Tukey’s HSD *post hoc* test (*P <* .05).

Changes in bacterial community composition reflected the differential responses of specific bacterial taxa to consumption by protists of different size categories. Among the top 10 bacterial phyla, *Bacteroidota* (ANOVA; *F* = 2.905, *P =* .048), *Planctomycetota* (ANOVA; *F* = 3.2608, *P =* .032), and *Verrucomicrobiota* (ANOVA; *F* = 3.7419, *P =* .019) were differentially affected by protist size categories. Specifically, Ps increased the relative abundance of *Bacteroidota* by 56% compared to P- (Tukey’s HSD, *P =* .045), Pm decreased the relative abundance of *Planctomycetota* by 67% compared to P- (Tukey’s HSD, *P =* .034) and Pl reduced the relative abundance of *Verrucomicrobiota* by 138%, compared to Ps (Tukey’s HSD, *P =* .020) (Fig. 2A).

Among the 40 most abundant bacterial genera, 15 showed significant responses to protist size categories (Fig. 2B). Several bacterial genera responded consistently across all protist size categories: *Flavobacterium* (ANOVA; *F* = 6.42, *P <* .01), *Neo-b11* (ANOVA; *F* = 5.66, *P <* 0.01), *Rugamonas* (ANOVA; *F* = 4.25, *P =* .011) and *Xylophilus* (ANOVA; *F* = 3.56, *P =* .023), decreased compared with P- (Tukey’s HSD, all *P <* .05), *Taonella* (ANOVA; *F* = 5.60, *P <* .01) and *Reyranella* (ANOVA; *F* = 8.22, *P <* .001) increased in relative abundance compared with P- (Tukey’s HSD, all *P <* .05). Other bacterial genera exhibited size-specific responses: *Pseudoflavitalea* (ANOVA; *F* = 7.30, *P <* .001) and *Hephaestia* (ANOVA; *F* = 10.59, *P <* .001) increased in relative abundance only in response to Ps compared with P-; *Streptomyces* (ANOVA; *F* = 2.59, *P =* .068) and *Micropepsaceae* (ANOVA; *F* = 5.09, *P <* .01) increased, while *Oligotropha* (ANOVA; *F* = 3.33, *P =* .030) decreased in relative abundance in response to Pl compared with P- (Tukey’s HSD, all *P <* .05; Fig. 2B).

LEfSe analysis further elucidated the specific bacterial taxa contributing significantly to the observed differences between protists of different size categories (Fig. 2C). Using an LDA threshold of 3.5, representative bacterial genera such as *env.OPS_17*, *Pseudoflavitalea*, *Hephaestia,* and *Taonella* were associated with Ps. Similarly, the *Bacteroidetes* bacterium was linked to Pm, while *Taibaiella*, *Micropepsaceae*, *Reyranella,* and *Kaistia* were representative genera for Pl (Fig. 2C).

Differential abundance analysis (ANCOM-BC2) also revealed both size-specific and common responses of bacterial taxa relative to P− (Fig. 2D). Some taxa responded uniquely to a single protist size category: the relative abundance of *Pseudomonas* decreased in Ps (lfc = −0.74, *P <* .05), *s3Psd-1089* decreased in Pm (lfc = −1.28, *P <* .05), *Isosphaeraceae* decreased in Pm (lfc = −1.35, *P <* 0.05), *Streptomyces* increased in Pl (lfc = 1.08, *P <* .05), and Nubsella decreased in Pl (lfc = −0.94, *P <* .05). Other taxa showed common responses across multiple protist size categories: the relative abundance of *Flavobacterium* decreased in Ps (lfc = −1.47), Pm (lfc = −2.18), and Pl (lfc = −2.08; all *P <* .05), while *Neo-B11* decreased in Ps (lfc = −1.01) and Pm (lfc = −1.28). In contrast, *Rhabdobacter* increased in Ps (lfc = 1.13), Pm (lfc = 1.11), and Pl (lfc = 0.94; all *P <* .05).

### Size effect of protists on litter mass loss

**Figure 3:**
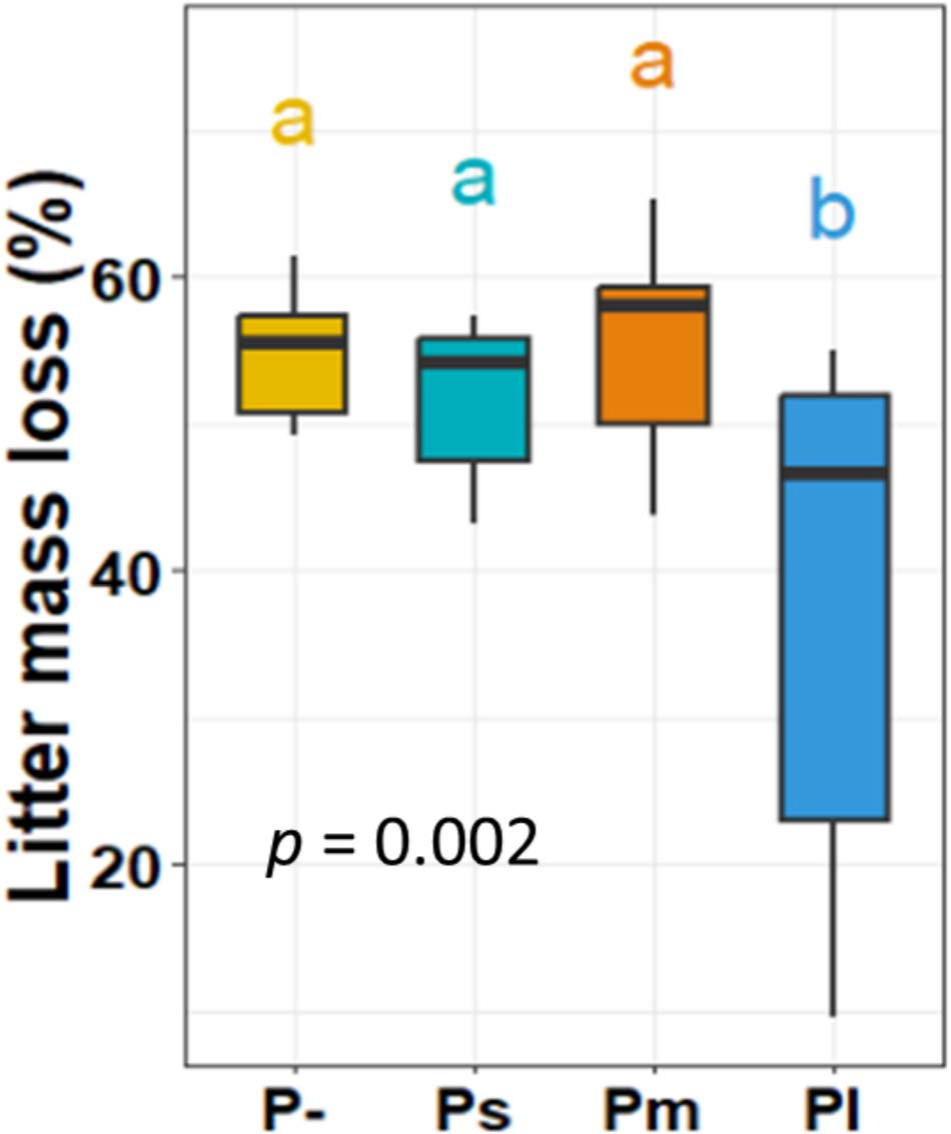
Size effects of protists on litter mass loss. P- = no-protist control, Ps = protists of the small-size category treatment, Pm = protists of the medium-size category treatment, Pl = protists of the large-size category treatment. Horizontal bars within boxes represent the median, with the tops and bottoms of boxes indicating the 75th and 25th quartiles, respectively. Whiskers depict the range of non-outlier data values, while outliers are plotted as individual points. Significant differences between different treatments are evaluated by a one-way ANOVA test (*P <* .05), with different letters above bars indicating significant distinctions tested by Tukey’s HSD *post hoc* tests (*P <* .05).

Litter mass loss was decreased by Pl (ANOVA; F = 5.96, *P =* .002; Fig. 3), while Ps and Pm did not significantly alter litter mass loss compared to P- (both Tukey’s HSD, *P* > .05). Specifically, Pl decreased litter mass loss by 40% compared to P- (Tukey’s HSD, *P <* .001), by 33% compared to Ps (Tukey’s HSD, *P =* .039), and by 44% compared to Pm (Tukey’s HSD, *P =* .003).

### Size effect of protists on microbial cumulative respiration

**Figure 4:**
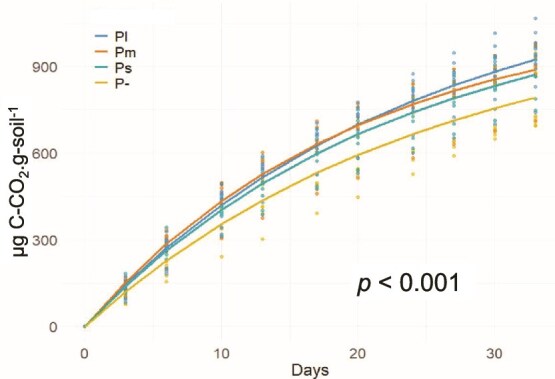
Size effects of protists on microbial cumulative respiration. Dots represent raw data, and solid lines show the best-fitting nonlinear mixed-effects model selected using Akaike information criterion (AIC). Unit = μg C-CO_2_.g-soil^−1^. P- = no-protist control, Ps = protists of the small-size category treatment, Pm = protists of the medium-size category treatment, Pl = protists of the large-size category treatment.

The best-fit nonlinear mixed-effects model showed that protist size influenced cumulative CO_2_ dynamics (likelihood ratio test: L.Ratio = 71.56, *P <* .0001). Two model parameters varied across protist size categories: ɸ_1_ (asymptote; total CO_2_ accumulated) and ɸ_3_ (rate constant; the rate of decline in respiration over time), while ɸ_2_ (initial respiration at Day 0) remained constant and unaffected by protist size. The parameter ɸ_1_ increased with increasing protist size, indicating greater total CO_2_ accumulation under larger protist treatments (Fig. 4). In contrast, ɸ_2_ was stable across all treatments, with an estimated value of −1.95. For ɸ_3_, the least negative value occurred under Pm (−2.980), compared to Ps (−3.126), Pl (−3.182), and P– (−3.233) (all *P*-values for Pm and Ps vs P– < 0.01; while Pl vs P– and Pl vs Ps > 0.05). This pattern suggests that respiration approached the asymptote most rapidly under Pm and Ps, but the trend did not follow a simple gradient with protist size (Fig. 4). Complementary repeated-measures ANOVA confirmed a time-dependent effect of protist size on cumulative respiration (interaction Time × Treatment: F = 3.25, *P <* .001; Fig. S6A). Pairwise contrasts showed that significant increases under Pl first appeared at Day 20 (*P =* .032) and persisted through Day 33, when Pl respiration was 17% higher than P− (*P =* .003). No significant differences were detected between Ps and Pm across time (all *P >* .05).

### Potential mechanism underlying the size effect of protists on litter decomposition

**Figure 5:**
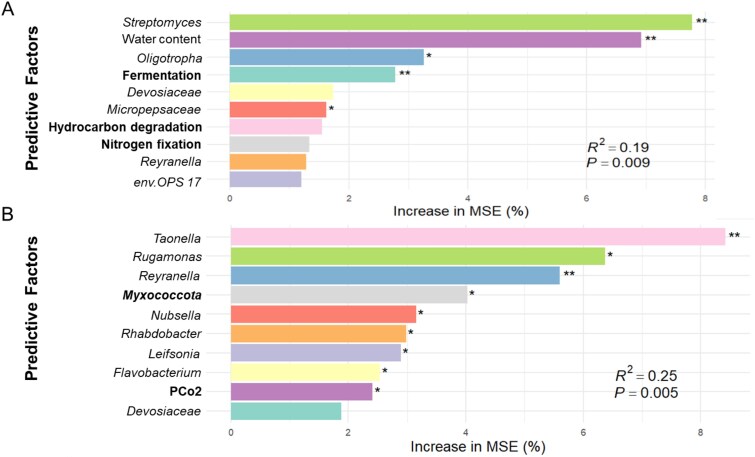
Potential drivers of variation in litter decomposition. Top 10 predictors obtained from a Random Forest (RF) analysis for litter mass loss (A) and cumulative respiration (B). Within the panels, bacterial genera are presented in italics, bacterial phyla are shown in bold italics, and physicochemical factors are displayed in regular font. FAPROTAX-annotated bacterial functional groups are indicated in bold. The accuracy importance measure was calculated for each tree and averaged across the forest (500 trees). Significances are labeled with asterisks (^*^P < .05; ^**^P < .01).

Random forest analysis identified *Streptomyces* (8%, *P <* .01), water content (7%, *P <* .01), *Oligotropha* (3%, *P =* .04), fermentation function (3%, *P <* .01), and *Micropepsaceae* (2%, *P =* .039) as key factors explaining variations in litter mass loss (Fig. 5A). Similarly, *Taonella* (8%, *P <* .01), *Rugamonas* (6%, *P =* .02), *Reyranella* (6%, *P <* .01), *Myxococcota* (4%, *P =* .02), *Nubsella* (3%, *P =* .02), *Rhabdobacter* (3%, *P =* .04), *Leifsonia* (3%, *P =* .03), and *Flavobacterium* (3%, *P =* .04) were identified as significant predictors of cumulative respiration (Fig. 5B).

## Discussion

Overall, our study provides new insights into the role of protist size in shaping bacterial communities and influencing litter decomposition, highlighting the importance of ecophysiological traits like protist size in modulating ecosystem functions.

Consistent with our first hypothesis and previous results for most protist species and communities tested [26–28], protists did not influence bacterial diversity, regardless of their size (Fig. 1A and B). These results may be explained by the fact that most protists either alone or in combination feed as generalists without fully eliminating specific bacterial taxa. Also in support of our first hypothesis, bacterial community dissimilarity increased with protist size category according to Bray–Curtis distance (Fig. 1C and D), supporting our first hypothesis. While protists of the small-size category (Ps) and protists of the medium-size category (Pm) did not differ from no-protist control (P-), protists of the large-size category (Pl) reduced Bray–Curtis distance relative to P-, Ps, and Pm (Fig. 1C), indicating that bacterial communities were more structurally similar in Pl than P-, Ps, and Pm. This is consistent with a previous study suggesting that protists of the large-size category exert stronger selective pressures on bacterial communities compared to protists of the small-size category and no-protist control [28]. Notably, our study using mixes of protists within the same size category expands the findings of Rocca *et al.* [28], which were based on single-species comparisons (*Tetrahymena pyriformis* as a protist of the small-size category and *Colpidium* sp. as a protist of the large-size category). Another study reported that, based on cell volume, protists of the large-size category made bacterial communities more structurally similar than protists of the smaller-size category did [39], supporting our results.

Despite these shifts in bacterial community composition, protist size categories did not alter the Gram-positive to Gram-negative ratio or the relative abundance of carbon-degrading functional groups (Figs S1–S2), contradicting this aspect of our first hypothesis. One possible explanation for this unexpected result is that the 35-day decomposition period might be insufficient to observe pronounced effects on bacterial functional processes [71]. Additionally, functional predictions based on FAPROTAX should be interpreted cautiously, as they rely on extrapolations from cultured organisms and assume functional consistency across taxa [72, 73]. Alternatively, rather than favoring Gram-positive bacteria, as initially expected, protists—particularly Pl—shifted bacterial communities in a Gram-independent manner toward other likely predation-resistant taxa such as *Streptomyces*, *Micropepsaceae*, *Taonella,* and *Reyranella* (Fig. 2B)*.* This suggests that traits beyond Gram classification influence bacterial resistance to protist predation, such as metabolic adaptations and morphological defenses [29]. For instance, the protist-induced increase in *Streptomyces* aligns with previous findings [74] and can be explained by its production of vast secondary metabolites, which enable superior growth of bacteria conducive to predation [75, 76]. Similarly, *Micropepsaceae, Taonella,* and *Reyranella*, members of *Alphaproteobacteria*, became more abundant with Pl, potentially by evading digestion through establishing symbioses with protists. For example, *Reyranella* has been documented to inhabit protist food vacuoles [77]. Additionally, some taxa among *Alphaproteobacteria* and *Gammaproteobacteria* can resist protist digestion through types III, IV, and VI secretion systems, suppressing protist defense mechanisms and facilitating intracellular survival [78–80].

In support of our second hypothesis, Pl reduced litter mass loss, whereas Ps and Pm had no measurable effect. This finding aligns with our expectation that protist effects increase with size category, as well as with previous research showing that smaller protists, such as *Didymium* sp. and *Cercomonas ambigua*, did not influence litter decomposition [38]. However, contrary to our second hypothesis, Pl increased microbial respiration (Fig. 3), especially in later phases, yet this was accompanied by reduced litter mass loss. One possible explanation is that bacteria under predation pressure preferentially used more readily available nitrogen-rich resources to proliferate (e.g. microbial necromass and metabolic byproducts), which are more labile and have a lower C:N ratio than our relatively recalcitrant litter material (C:N ≈ 32) [81]. Such a shift would increase microbial respiration but reduce direct litter decomposition. In addition, the accelerated respiration under Pl may also reflect priming effects on SOM, as shown by Olayemi *et al.* [82], where the addition of lactobionate stimulated microbial respiration from native SOM in excess of the lactobionate-derived CO_2_. Together, these mechanisms may explain the observed discrepancy between respiration and litter mass loss, though direct evidence supporting these mechanisms still needs to be collected in future studies.

The observed decrease in litter mass loss alongside increased microbial respiration under Pl can partially be explained by shifts in bacterial community composition associated with protist size category, as revealed by our random forest analysis, supporting our third hypothesis. Previous studies have shown that protist-induced changes in bacterial communities can influence ecosystem functions, such as plant growth, by promoting predation-resistant bacteria [74], though similar effects on litter decomposition remain largely unexplored. In our study, shifts in bacterial community composition—such as a decline in *Oligotropha,* a genus of oligotrophic heterotrophs that utilize low-nutrient organic carbon sources [16], and an increase in *Streptomyces*, a genus known to resist protist predation and inhibit fungi and other bacteria [24, 74]—suggest potential links between protist-induced bacterial community shifts and litter decomposition. Similarly, shifts in bacterial community composition from likely easily digestible *Nubsella* and *Rugamonas* to potential protist symbionts (*Taonella* and *Reyranella*), which possess efficient protein secretion systems [78, 79], suggest a connection between protist-induced bacterial shifts and microbial respiration. According to Grime’s triangle theory, bacteria under strong predation pressure—such as by Pl—may shift their functional strategies toward defense-oriented traits, including increased extracellular protein production to resist digestion [83, 84]. While these shifts may influence bacterial metabolism, symbionts represent only a small fraction of the bacterial community, limiting their direct contribution to overall microbial respiration. Generally, bacterial community changes alone explain only a small portion of litter mass loss and microbial respiration (Fig. 5), suggesting that additional factors, such as fungal activity and microbial-derived carbon accumulation (e.g. microbial necromass) [85], may also contribute to regulating litter decomposition.

## Conclusion

In conclusion, protist communities, particularly Pl, restructured bacterial communities into a more structurally similar assemblage dominated by likely predation-resistant bacteria. These shifts explained reduced litter mass loss and increased microbial respiration, which were not observed in other protist size categories (Ps and Pm). The finding that protist size-induced differences might determine litter decomposition was derived from a robust, novel experimental design. The 12 protist species were grouped into three size categories, with replicates consisting of different species assemblies, providing a species-independent overview of protist size effects. This study provides the foundation for a better understanding of litter decomposition, the carbon cycle, and the role of protists therein. We acknowledge that we cannot directly observe protist predation in our experimental setup using opaque soil and litter. Future studies setting up similar experiments should expand our findings and determine the specific flow of nutrients, particularly carbon and nitrogen, from microbes to protists, such as by applying stable isotope probing [48]. In addition, we recommend expanding our trait range by including a broader diversity of protist species that extends the size categories studied here and including additional life-history traits differentiating protists (e.g. habitat preference, motility; [86, 87]) to reveal their differential roles in soil food webs. Building on body size effects observed here, future studies could also combine protists from different size categories to evaluate whether mixed-size communities exploit a broader prey spectrum and strengthen predation effects. Finally, we recommend extended time scales to evaluate shifts in predator–prey dynamics over time and potential taxonomic, functional, and trait-specific differences during decomposition [88].

## Supplementary Material

Supplementary_Information_ycaf231

## Data Availability

The raw 16S rRNA bacterial sequences have been deposited in the Sequence Read Archive (SRA) at the National Center for Biotechnology Information (NCBI) under BioProject accession number PRJNA1245502. Analysis scripts and processed data have been deposited on GitHub (https://github.com/YX17Wang/PSLD.git).
