# Supplementary material for: Protist size-dependent shifts of bacterial communities can reduce litter decomposition
Source: ISME Commun. 2025 Dec 6;5(1):ycaf231. doi: 10.1093/ismeco/ycaf231 (PMC12753304; doi:10.1093/ismeco/ycaf231)
Supplement: Supplementary_Information_ycaf231 [file supplementary_information_ycaf231.pdf]

SUPPLEMENTARY INFORMATION

1. Supplementary material and methods

1.1 Protist inoculum preparation

Cultures of 12 protist species were used, with more information provided in Supplementary Table S1. These protist species were extracted as described in Berlinches de Gea et al. [1]. These protist species were selected because they represent widespread bacterivorous taxa commonly occurring in soils and litter residues, spanning different taxonomic groups and body sizes. By including species from different lineages within each size category, we reduced the potential influence of lineage-conserved traits (e.g., enzymes) on the observed outcomes. Twenty days before inoculation, ten Petri dishes (50 x 15 mm) (Greiner Bio-One, The Netherlands) were prepared for each protist species. Each Petri dish was inoculated with 5 mL of 9:1 NMAS: NB-NMAS medium and 150 μL of stock protist species. These dishes were placed in a temperature-controlled dark incubator (Hettich, Germany) at 16°C for 20 days. After incubation, we mixed the contents of five Petri dishes (2 Falcon tubes for each protist). Then, we centrifuged protists inocula at 1,200 rpm for 5 min using a Heraeus Multifuge 3S Centrifuge (Heraeus, US). Afterward, we discarded the 15 mL supernatant until 10 mL remained and added 10 mL NMAS to wash them three times. Finally, we pooled the contents of the two tubes together for each protist species. We then counted protist individuals using a Zeiss Axioskop 2 Plus microscope (Zeiss, Germany) at 200x magnification and diluted them in NMAS to the concentration of 10 protists per uL. Exceptions were made for *Rosculus sp.* (C10D3) from protists of the small-size category, Cercozoa (2M) from protists of the medium-size category, and *Vannella* sp. (P147) from protists of the large-size category, which were adjusted to a concentration of 2 or 3 protists per μL due to their lower concentration compared to other cultures (see Table S2 for details of the inoculation scheme). Although the initial concentration

of protists might not be critical since the inoculated protists were allowed to establish in the soil for one week, we still analyzed the data afterwards and verified that these three protist species with lower initial abundances did not significantly deviate from the average effects of the other cultures on overall litter mass loss, soil respiration, and bacterial alpha diversity and composition (Fig. S3-5).

1.2 Bacterial inoculum preparation

Bacterial cultures originated from meadow (51°58′23″ N 5°43′05″ E) and garden (51°58′18″ N 5°41′02″ E) soil in the Netherlands. These bacterial cultures were extracted as described in Berlinches de Gea et al. [1]. These soils for bacterial communities extraction were chosen because they represent typical temperate agroecosystem habitats with high bacterial diversity, which represent the main colonizers in litter residues. We prepared eight 50 mL Falcon tubes for bacterial inoculum cultivation one day before inoculation. Each tube was inoculated with 20 mL Lysogeny broth (LB) (1 L demineralized water with 10 g bacterial peptone (OXOID, UK), 10 g NaCl (DUCHE, The Netherlands) and 5 g yeast extract (OXOID, UK)) and 50 µL bacterial inoculum. We left the screw cap of these Falcon tubes slightly loose to support the growth of both aerobic and anaerobic bacteria. We incubated these tubes at 250 rpm in an Innova 4330 incubator shaker (New Brunswick Scientific, New Jersey) at 30 °C for 24 h. After incubation, we centrifuged the bacterial inoculum using the Heraeus Multifuge 3S Centrifuge (Heraeus, US) at 3,000 rpm for 15 min. The supernatant was discarded. To remove access nutrients, we washed the bacterial pellet twice with 20 mL of Neff's Modified Amoebae Saline (NMAS) [2] by centrifuging the inoculum at 1,500 rpm for 5 min each time. Subsequently, we resuspended the pellet in 5 mL NMAS and vortexed the tubes (Vortex-Genie 2; Scientific Industries, Inc., United States). The contents of eight tubes were combined into one, resulting in a final volume of 40 mL.

1.3 Fungal inoculum preparation

Cultures of 13 fungal species (*Acremonium* sp., *Alternaria* sp., *Arthrinium* sp., *Chaetomium* sp., *Fusarium oxysporum, Fusarium avenaceum, Ilyonectria* sp., *Mucor* sp., *Neonetrica* sp., *Penicillium* sp., *Plectosphaerella* sp., *Tetracladium* sp. *and Trichoderma* sp.) were used, with more details on sampling, isolation and culturing provided in Geisen et al. [3]. These fungal species were selected because they are commonly found in arable soils and litter residues and are (facultative) saprotrophs as grown on agar. Ten days before inoculation, these fungal species were sub-cultured on potato dextrose agar media (PDA) in Petri dishes (90 x 15 mm) (Greiner Bio-One, The Netherlands) [4]. Then, we cut a 5 mm diameter plug from stock fungal species using a sterilized blade and placed it centrally on the solidified PDA agar plate (90 x 15 mm) (Greiner Bio-One, The Netherlands). These newly cultivated fungal species were incubated in an Agilent Hybridization Oven (Agilent Technologies, United States) at 25 °C for ten days. After incubation, we used a cell scraper (Greiner Bio-One B.V., Kremsmünster) to remove the surface mycelium or spores from the Petri dishes, transferring them into prepared 50 mL Falcon tubes. Four Falcon tubes were filled with the fungal suspension. The tubes were then centrifuged at 3,000 rpm for 15 min. After centrifugation, we carefully discarded 30 mL of the supernatant, leaving 10 mL of concentrated fungal suspension in each tube. We washed the fungal suspension twice with 20 mL NMAS by centrifuging the inoculum at 1,500 rpm for 5 min to remove access nutrients. Following the washes, the upper layer was discarded, and 10 mL of fungal suspension was retained. All fungal suspensions were pooled together, resulting in a final volume of 40 mL.

2. Supplementary references

1. Berlinches de Gea A, Li G, Chen J, Wu W, Kohra A, Aslan SK, et al. Increasing soil protist diversity alters tomato plant biomass in a stress-dependent manner. *Soil Biol Biochem* 2023; **186**: 109179.

2. Page FC. An illustrated key to freshwater and soil amoeba, with notes on cultivation and ecology. *Fresh Boil Ass Sci Publ* 1976; **34**: 1–155.

3. Geisen S, ten Hooven FC, Kostenko O, Snoek LB, van der Putten WH. Fungal root endophytes influence plants in a species-specific manner that depends on plant's growth stage. *J Ecol* 2021; **109**: 1618–1632.

4. Shim SM, Oh YH, Lee KR, Kim SH, Im KH, Kim JW, et al. The characteristics of cultural conditions for the mycelial growth of *Macrolepiota procera*. *Mycobiology* 2005; **33**: 15–18.

5. Gao Z. Soil protists: from traits to ecological functions (Doctoral Dissertation). 2020. Utrecht University.

3. Supplementary tables and figures

Table S1 Protists list used in this experiment and their corresponding size information. The values are presented as means ± standard errors (SEs). Cyst volume (µm³): calculated as the volume of a sphere using the formula $\frac{4}{3}\pi r^3$. Active protist volume (µm³): calculated based on the morphotype group of protists using different geometric formulas [5] of each protist based on measurements obtained from at least ten individuals per species. The table is organized by size categories using Method 4, which categorizes protists based on the area measurement method.

Code	Name	Supergroup	Morphotype Group	Method 1	Method 2	Method 3	Method 4	Size categories
				**Cyst volume (µm³)**	**Maximum length (µm)**	**Active protist volume (10³ µm³)**	**Protist area (10³ µm²)**	

C10D3	*Rosculus* sp.	TSAR	B	320.59 41.80	±91.08 4.23	±141.55 ± 14.33	5.65 ± 0.35	Small
19M	Cercozoa	TSAR	B	4,057.75 386.68	±121.57 3.82	±407.43 ± 26.68	11.11 ± 0.41	Small
1-1	*Didymium* sp.	Amorphea	B	223.10 23.14	±162.29 8.23	±562.14 ± 51.05	15.09 ± 0.94	Small
S18D10	*Heterolobosea*	Excavates	A	1,720.29 211.79	±187.10 15.28	±1,535.81 322.37	±18.72 ± 2.58	Small
2M	Cercozoa	TSAR	B	2,039.06 203.34	±231.81 14.50	±1,483.83 165.69	±28.46 ± 1.73	Medium
Protist10	*Allovahlkampfia* sp.	Excavates	A	2,385.79 233.06	±280.72 19.71	±6,411.26 766.85	±47.27 ± 4.37	Medium
33	*Heterolobosea*	Excavates	B	5,722.18 1375.15	±363.52 15.94	±2,896.68 246.46	±50.78 ± 2.6	Medium
C13D2	*Acanthamoeba* sp.	Amorphea	C	4,870.54 928.11	±296.49 19.41	±32.12 ± 3.08	64.23 ± 6.17	Medium
P2881	*Naegleria* sp.	Excavates	A	5,071.57 485.61	±387.51 19.34	±9,542.83 896.46	±68.01 ± 4.19	Large
S28D2	*Allovahlkampfia* sp.	Excavates	A	3,772.41 311.64	±349.95 15.51	±11,918.89 2,259.05	±69.73 ± 7.38	Large
75	*Cryptodifflugia* sp	Amorphea	A	21,247.33 ± 2,734.22	295.94 9.07	±16,289.81 792.77	±78.25 ± 2.97	Large
P147	*Vannella* sp.	Amorphea	C	4,822.63 575.17	±312.11 24.21	±43.89 ± 7.01	87.78 ± 14.01	Large

Table S2 Protists inoculation scheme. Ps referred to protists of the small-size category treatment, Pm referred to protists of the medium-size category treatment, and Pl referred to

protists of the large-size category treatment; values are the total inoculated number of protists in each pot. Most protist cultures were standardized to a concentration of 10 protists/μL. Exceptions included *Rosculus* sp. (C10D3) from the Ps treatment and Cercozoa (2M) from the Pm treatment, which were adjusted to 2 protists/μL, and *Vannella* sp. (P147) from the Pl treatment, which was adjusted to 3 protists/μL due to their lower culture concentrations. Each protist species was inoculated into the pots at a volume of 1 mL.

	Ps				Pm				Pl			
Code	C10D3	19M	1-1	S18D10	2M	33	Protist10	C13D2	P2881	75	S28D2	P147
Treatment	*Rosculus* sp.	Cercozoa	*Didymium* sp.	*Heterolobosea*	Cercozoa	*Heterolobosea*	*Allovahlkampfia* sp.	*Acanthamoeba* sp.	*Naegleria* sp.	*Cryptodifflugia* sp.	*Allovahlkampfia* sp.	*Vannella* sp.
Ps	2,000	10,000	10,000									
Ps	2,000	10,000		10,000								
Ps	2,000		10,000	10,000								
Ps		10,000	10,000	10,000								
Ps	2,000	10,000	10,000									
Ps	2,000	10,000		10,000								

Ps	2,000		10,000	10,000				
Ps		10,000	10,000	10,000				
Ps	2,000	10,000	10,000					
Ps	2,000	10,000		10,000				
Pm					2,000	10,000	10,000	
Pm					2,000	10,000		10,000
Pm					2,000		10,000	10,000
Pm						10,000	10,000	10,000
Pm					2,000	10,000	10,000	
Pm					2,000	10,000		10,000
Pm					2,000		10,000	10,000
Pm						10,000	10,000	10,000

Pm	2,000	10,000	10,000					
Pm	2,000	10,000		10,000				
Pl					10,000	10,000	10,000	
Pl					10,000	10,000		3,000
Pl					10,000		10,000	3,000
Pl						10,000	10,000	3,000
Pl					10,000	10,000	10,000	
Pl					10,000	10,000		3,000
Pl					10,000		10,000	3,000
Pl						10,000	10,000	3,000
Pl					10,000	10,000	10,000	
Pl					10,000	10,000		3,000

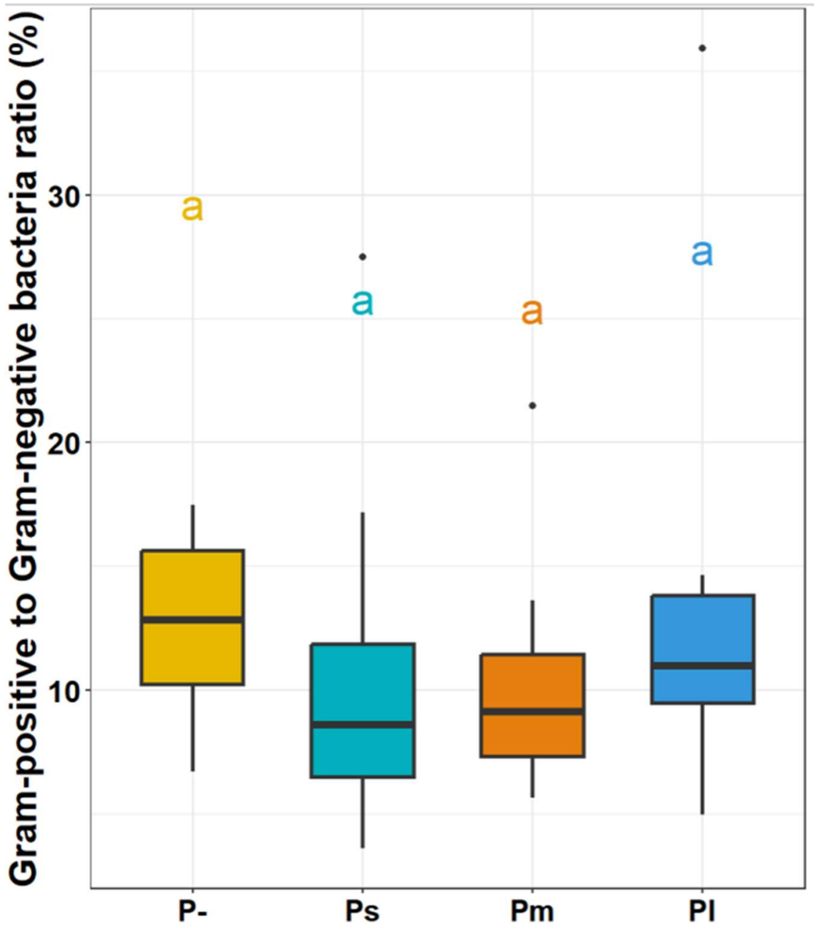

Fig. S1. Size effect of protists on the Gram-positive to Gram-negative bacteria ratio based on all bacterial phyla. We classified the phyla *Actinobacteriota* and *Bacillota* as Gram-positive bacteria, whereas *Abditibacteriota*, *Acidobacteriota*, *Armatimonadota*, *Bacteroidota*, *Bdellovibrionota*, *Chloroflexota*, *Cyanobacteriota*, *Thermodesulfobacteriota*, *Gemmatimonadota*, *Myxococcota*, *Planctomycetota*, *Pseudomonadota,* and *Verrucomicrobiota* were classified as Gram-negative bacteria. P- = no-protist control, Ps = protists of the small-size category treatment, Pm = protists of the medium-size category treatment, Pl = protists of the large-size category treatment. Horizontal bars within boxes represent the median, with the tops and bottoms of boxes indicating the 75th and 25th quartiles, respectively. Whiskers depict the range of non-outlier data values, while outliers are plotted as individual points. Significant

differences between different treatments are evaluated by a Kruskal-Wallis test, with different letters above bars indicating significant distinctions tested through Dunn's test ($p < 0.05$).

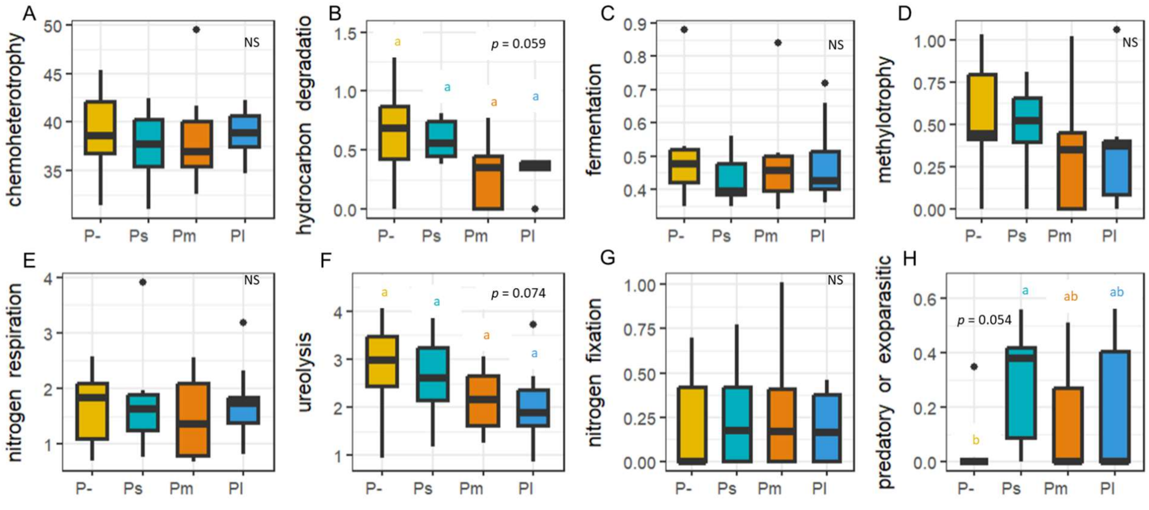

Fig. S2. Size effect of protists on the relative abundance of FAPROTAX-annotated bacterial functional groups. Effects of protist size on bacterial functional groups related with carbon cycling (A-D) and nitrogen cycling (E-G) and predatory bacteria group (H). P- = no-protist control, Ps = protists of the small-size category treatment, Pm = protists of the medium-size category treatment, Pl = protists of the large-size category treatment. Horizontal bars within boxes represent the median, with the tops and bottoms of boxes indicating the 75th and 25th quartiles, respectively. Whiskers depict the range of non-outlier data values, while outliers are plotted as individual points. In panels B and F, significant differences between different treatments are evaluated by a one-way ANOVA test ($p < 0.05$), with different letters above bars indicating significant distinctions tested through Tukey's HSD post-hoc test ($p < 0.05$). In panel H, important differences between different treatments are evaluated by a Kruskal-Wallis test, with different letters above bars indicating significant distinctions tested through Dunn's test ($p < 0.05$). NS means no significance.

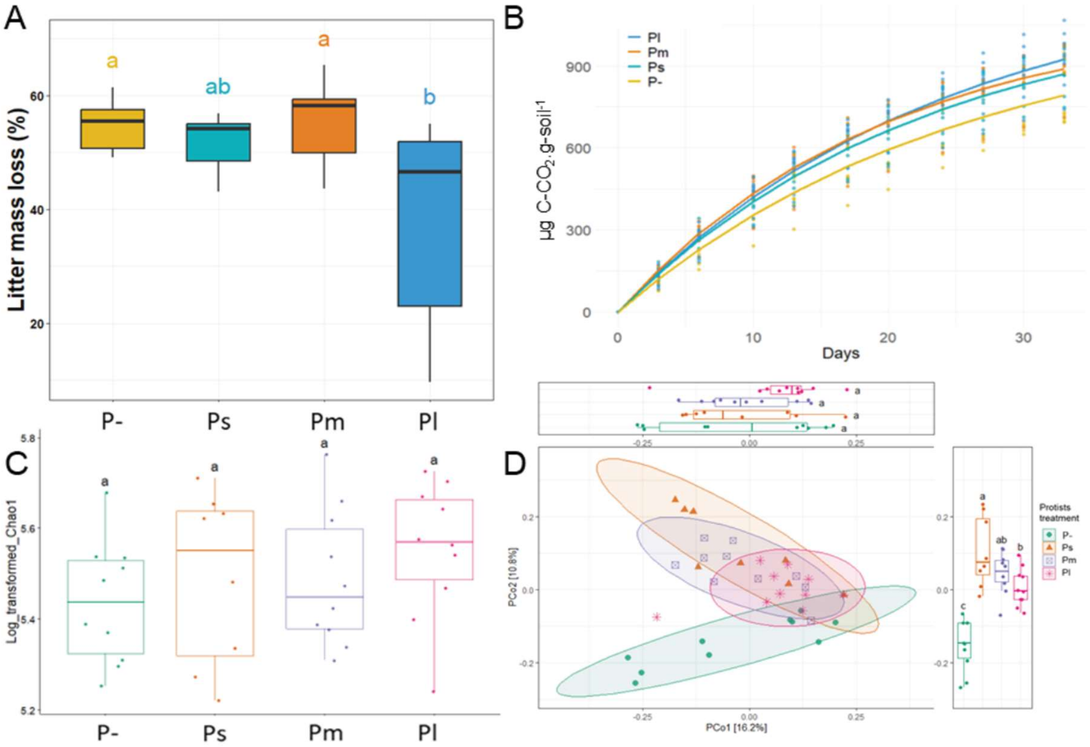

Fig. S3. Size effect of protists (excluding *Rosculus* sp. (C10D3) from Ps) on litter decomposition, microbial respiration and bacterial communities. Effects of protist size (excluding *Rosculus* sp. (C10D3) from Ps) on litter mass loss (%) (A), cumulative carbon loss via respiration (μg $C\text{-}CO_2.g\text{-}soil^{-1}$) (B), α-diversity Chao1 (C) and β-diversity of bacterial community analyzed via Principal Coordinate Analysis (PCoA) (D). P- = no-protist control, Ps = protists of the small-size category treatment, Pm = protists of the medium-size category treatment, Pl = protists of the large-size category treatment. In panels A, C, and D, horizontal bars within boxes represent the median, with the tops and bottoms of boxes indicating the 75th and 25th quartiles, respectively. Whiskers depict the range of non-outlier data values, while outliers are plotted as individual points. Significant differences between different treatments are evaluated by a one-way ANOVA test ($p < 0.05$), with different letters above bars indicating significant distinctions tested through Tukey's HSD post-hoc test ($p < 0.05$). In panel B, dots

represent raw data, and solid lines show the best-fitting nonlinear mixed-effects model selected using Akaike Information Criterion (AIC).

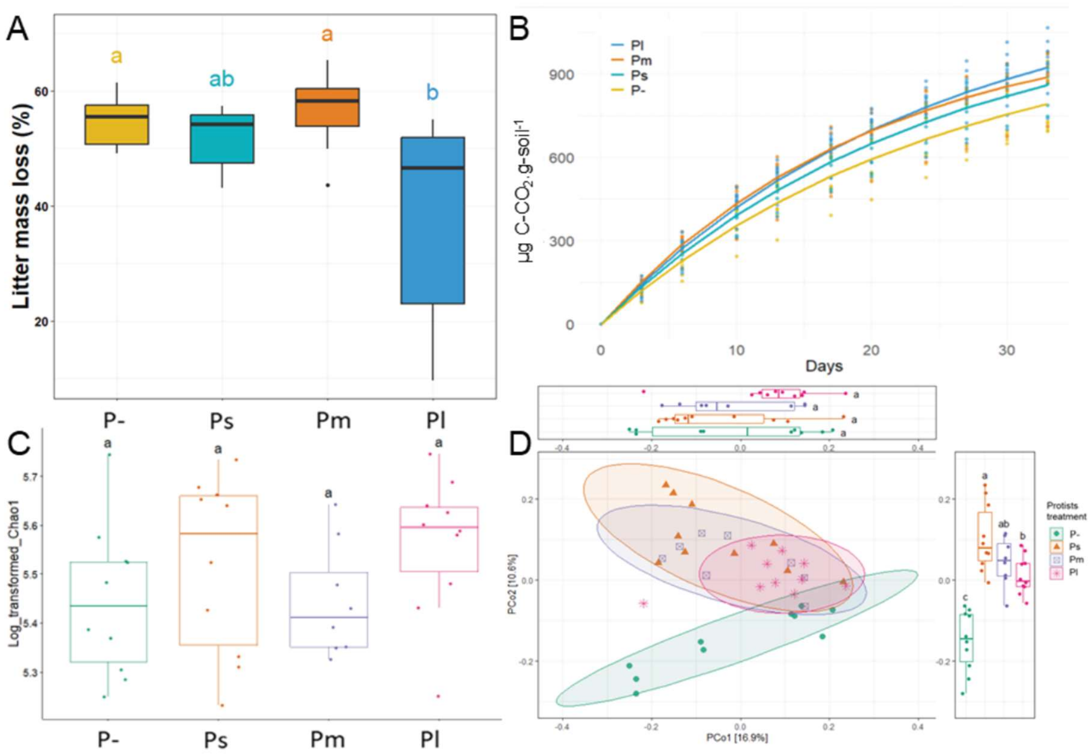

Fig. S4. Size effect of protists (excluding Cercozoa (2M) from Pm) on litter decomposition, microbial respiration and bacterial communities. Effects of protist size (excluding Cercozoa (2M) from Pm) on litter mass loss (%) (A), cumulative carbon loss via respiration (μg C-CO_2.g-soil^{-1}) (B), α-diversity Chao1 (C) and β-diversity of bacterial community analyzed via Principal Coordinate Analysis (PCoA) (D). P- = no-protist control, Ps = protists of the small-size category treatment, Pm = protists of the medium-size category treatment, Pl = protists of the large-size category treatment. In panels A, C, and D, horizontal bars within boxes represent the median, with the tops and bottoms of boxes indicating the 75th and 25th quartiles, respectively. Whiskers depict the range of non-outlier data values, while outliers are plotted as individual points. Significant differences between different treatments are evaluated by a one-way ANOVA test ($p < 0.05$), with different letters above bars indicating significant distinctions

tested through Tukey's HSD post-hoc test ($p < 0.05$). In panel B, dots represent raw data, and solid lines show the best-fitting nonlinear mixed-effects model selected using Akaike Information Criterion (AIC).

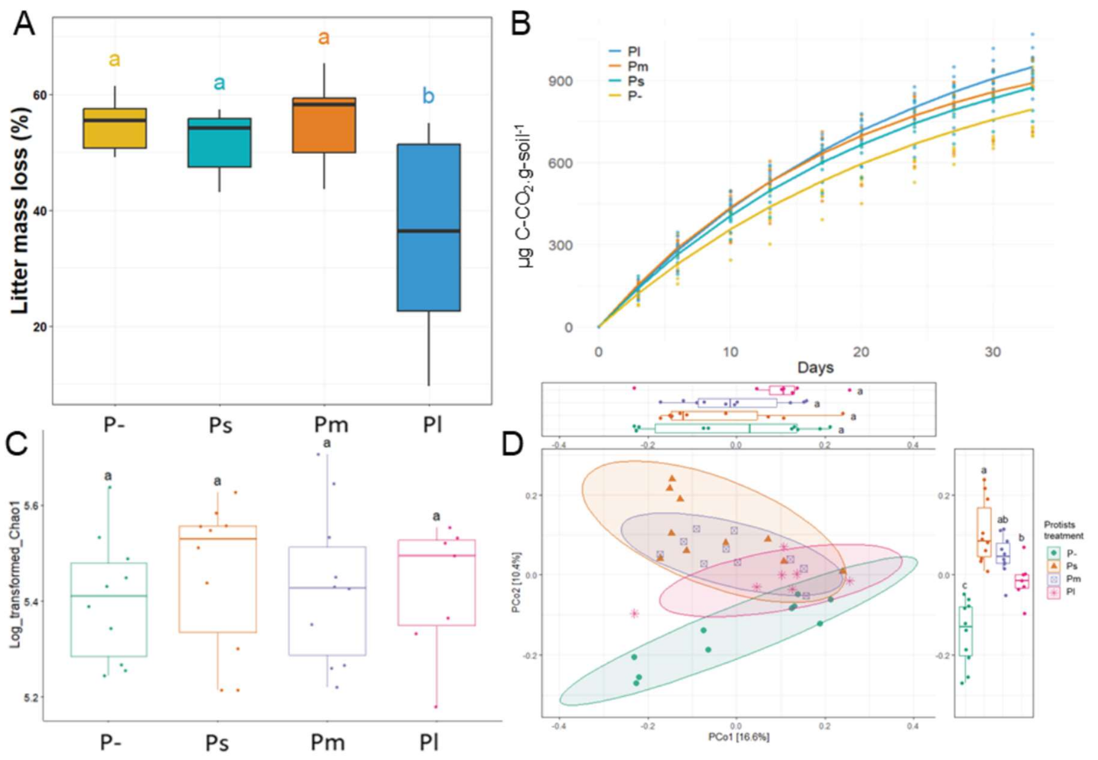

Fig. S5. Size effect of protists (excluding *Vannella* sp. (P147) from Pl) on litter decomposition, microbial respiration and bacterial communities. Effects of protist size (excluding *Vannella* sp. (P147) from Pl) on litter mass loss (%) (A), cumulative carbon loss via respiration (µg C-CO_2.g-soil^{-1}) (B), α-diversity Chao1 (C) and β-diversity of bacterial community analyzed via Principal Coordinate Analysis (PCoA) (D). P- = no-protist control, Ps = protists of the small-size category treatment, Pm = protists of the medium-size category treatment, Pl = protists of the large-size category treatment. In panels A, C, and D, horizontal bars within boxes represent the median, with the tops and bottoms of boxes indicating the 75th and 25th quartiles, respectively. Whiskers depict the range of non-outlier data values, while outliers are plotted as individual points. Significant differences between different treatments are

evaluated by a one-way ANOVA test ($p < 0.05$), with different letters above bars indicating significant distinctions tested through Tukey's HSD post-hoc test ($p < 0.05$). In panel B, dots represent raw data, and solid lines show the best-fitting nonlinear mixed-effects model selected using Akaike Information Criterion (AIC).

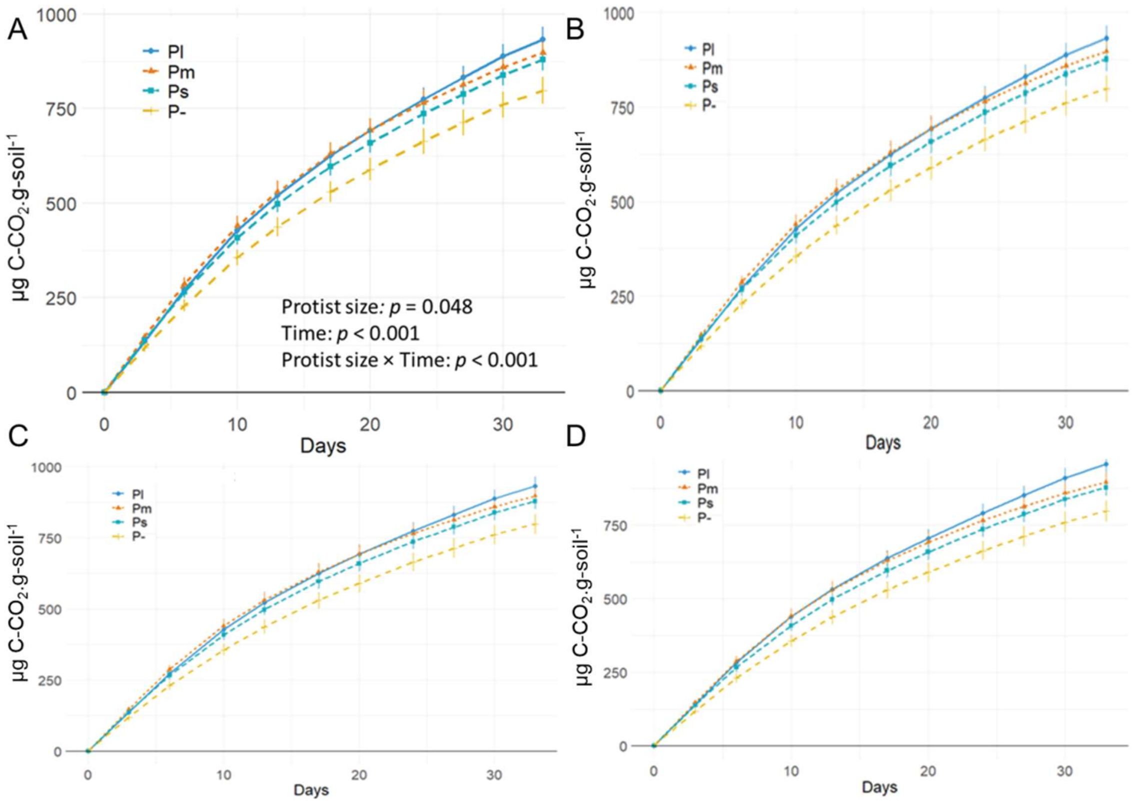

Fig. S6. Size effects of protists on microbial cumulative respiration. Panels: (A) all samples included; (B) excluding *Rosculus* sp. (C10D3) from Ps; (C) excluding Cercozoa (2M) from Pm; (D) excluding *Vannella* sp. (P147) from Pl. Unit = μg C-CO_2.g-soil^{-1}. P- = no-protist control, Ps = protists of the small-size category treatment, Pm = protists of the medium-size category treatment, Pl = protists of the large-size category treatment. Values are means ± SEs (n = 10). Repeated-measures ANOVA tested the effects of protist size, time, and their interaction on microbial cumulative respiration ($p < 0.05$).
